# Unintended Genomic Outcomes in Current and Next Generation GM Techniques: A Systematic Review

**DOI:** 10.3390/plants11212997

**Published:** 2022-11-07

**Authors:** Philomena Chu, Sarah Zanon Agapito-Tenfen

**Affiliations:** NORCE Norwegian Research Centre AS, Climate & Environment Division, Siva Innovasjonssenter, Sykehusvn 21, 9019 Tromsø, Norway

**Keywords:** genetically modified organisms, transgenic, CRISPR, gene-editing, biosafety, sequencing

## Abstract

Classical genetic engineering and new genome editing techniques, especially the CRISPR/Cas technology, increase the possibilities for modifying the genetic material in organisms. These technologies have the potential to provide novel agricultural traits, including modified microorganisms and environmental applications. However, legitimate safety concerns arise from the unintended genetic modifications (GM) that have been reported as side-effects of such techniques. Here, we systematically review the scientific literature for studies that have investigated unintended genomic alterations in plants modified by the following GM techniques: *Agrobacterium tumefaciens*-mediated gene transfer, biolistic bombardment, and CRISPR-Cas9 delivered via *Agrobacterium*-mediated gene transfer (DNA-based), biolistic bombardment (DNA-based) and as ribonucleoprotein complexes (RNPs). The results of our literature review show that the impact of such techniques in host genomes varies from small nucleotide polymorphisms to large genomic variation, such as segmental duplication, chromosome truncation, trisomy, chromothripsis, breakage fusion bridge, including large rearrangements of DNA vector-backbone sequences. We have also reviewed the type of analytical method applied to investigate the genomic alterations and found that only five articles used whole genome sequencing in their analysis methods. In addition, larger structural variations detected in some studies would not be possible without long-read sequencing strategies, which shows a potential underestimation of such effects in the literature. As new technologies are constantly evolving, a more thorough examination of prospective analytical methods should be conducted in the future. This will provide regulators working in the field of genetically modified and gene-edited organisms with valuable information on the ability to detect and identify genomic interventions.

## 1. Introduction

Instilled in many of us is the desire to understand the phenomena around us and sometimes to control these phenomena. One of the best known examples of this is the domestication of teosinte into the corn that we know today. Ancient plant breeders some 10,000 years ago in Mexico started selectively breeding teosinte for more desirable characteristics, such as larger cobs and more kernels. The constant drive to shape organisms’ physiology to our needs and desires forever altered our relationship with other species. Modern day technologies, such as genetic engineering, further changed the way we interact with organisms. Yet, our understanding of these technologies and their repercussions as they ripple throughout the natural world is still lacking. Many of the biotechnology tools employed in genetic engineering and genome editing are systems taken from nature and tweaked to serve our purposes. This often results in an incomplete understanding of aspects of the system’s functioning, which are not entirely within our control, leading to unintended consequences [1].

In plants, *Agrobacterium tumefaciens*-mediated gene transformation and biolistic bombardment are the main ways to introduce foreign DNA into plants and have been used for decades. By now, many economically important crops have been transformed using these two technologies. In terms of cultivated area, 92 Mhas of transgenic soybean and 62 Mhas of transgenic maize, both being transformed using either *Agrobacterium* or biolistics, respectively, have been planted worldwide [2]. In ISAAA’s GM approval database, 214 events transformed by *A. tumefaciens* and 45 biolistic bombardment events have been approved [3].

Owing to advances in gene and genome sequencing and the development of gene editing technologies, more precise and targeted edits were made possible and with greater speed. However, technologies like zinc-finger nucleases (ZFNs) and transcription activator-like effector nucleases (TALENs) involve the building and engineering of modular proteic units and are therefore costly compared to the simplicity of CRISPR system where the CRISPR associated protein 9 (Cas9) enzyme is complexed with a guide RNA designed to target the gene(s) or locus/loci. CRISPR-Cas technology has been hailed as “targeted,” “precise,” “safe” and “simple” [4,5,6]. However, research has demonstrated that the genetic outcome of using this technology is not a priori reliably predictable. In SDN1 situations, the double-stranded break is repaired via the cellular machinery, but there is little to no control over the sequences that are introduced at the repair site. Evidence of off-targeting and unintended consequences in plants resulting from CRISPR-Cas have been extensively discussed in the literature [7,8]. DNA edits at off-target sites could potentially lead to unintended genotypic and phenotypic outcomes.

Despite the widespread use of genetically modified organisms, the need for biosafety evaluation remains a concern, and it is mandated in the domestic legislation of many countries as well as in international treaties [9,10]. Such regulations demand pre-market risk assessment to evaluate any risks that GM plants may pose to animal and human health and the environment. As part of the pre-market risk assessment, many regulatory authorities evaluate all relevant scientific data on the molecular characterization of the GM plant in question, such as the source and function of the donor DNA, the transformation method, the organization of the inserted DNA at the insertion site(s), and the expression and stability of the insert [11,12]. In July 2018, the European Union’s Court of Justice ruled that gene-edited organisms should be regulated as genetically modified organisms (GMOs). Therefore, it is also relevant to review the available empirical data on the unintended genomic changes as consequences of such new technologies and also the analytical methods applied to those studies.

In this context, we have chosen five methods of genetic modification for this study—*Agrobacterium tumefaciens*-mediated gene transformation, biolistics/biolistic bombardment, stable expression of CRISPR-Cas9 via *Agrobacterium*-mediated transformation and biolistics, and transient expression with ribonucleoproteins (RNPs)—based on their popularity amongst researchers, their relevance in the current landscape and focused on recent advances in gene technology.

First, we focus on transformation with *Agrobacterium tumefaciens*, the causal agent of crown gall disease*. Agrobacterium*-mediated transformation is a biological process that involves complex bacteria and host interactions. *A. tumefaciens* contains a Ti (tumor-inducing) plasmid that contains a T-DNA (transfer DNA) region, flanked by two 25 bp direct repeats referred to as left and right border sequences. The sequence of this T-DNA region can be swapped out to contain a DNA sequence-of-interest. Once activated, the T-DNA region is transferred from the Ti plasmid to the host cell and ultimately integrated into a random location in the host genome [13]. These days, *A. tumefaciens* transformation of many economically important crops has been established.

Biolistics or biolistic bombardment is a physical method of introducing foreign DNA into the plant genome, and can be adapted to introduce mRNA and protein. The DNA is coated onto gold or tungsten particles and then bombarded at plant tissue at high pressure. The DNA can directly enter the plant cell and then be integrated into the host genome. Unlike with *Agrobacterium*-mediated transformation, no binary vector is required. Naked DNA can be used. Since this is a physical method, there are no limitations to compatible plant species. It is possible to use various tissue and cell types with biolistic transformation. Some disadvantages that have been revealed over the years are that the gene gun used to perform the experiment is expensive, the force of the bombardment can cause tissue damage, the integration of the DNA may be complex [14,15]. Biolistic bombardment can also be used for chloroplast and mitochondria transformation, but we focus on nuclear plant transformation here.

We then shift our focus to CRISPR-Cas9 gene editing. The CRISPR-Cas9 (clustered regularly interspaced short palindromic repeat) system consists of a Cas9 DNA nuclease and a guide RNA (gRNA) that leads the Cas9 enzyme to a specified target location in the genome. Together, they generate double-stranded breaks at specific target sites. The break is then repaired either by homologous end joining (NHEJ) or homology-directed recombination (HDR). NHEJ is the predominant repair mechanism, but lacks the precision that HDR can produce. Consequently, NHEJ introduces short insertion or deletion mutations (INDELs) at the double-stranded breaks [16,17,18]. Thorough examination of on-target and off-target consequences resulting from CRISPR edits will be necessary for future development of this technology in plants.

There are multiple ways to deliver the CRISPR-Cas9 reagents into plant cells. With *A. tumefaciens* delivery, Cas9 and gRNA are introduced as DNA through expression cassettes in the T-DNA. The *Agrobacterium* transfers the T-DNA into the host cell, then the CRISPR reagents are expressed thereby producing genome edits. As is the case with *Agrobacterium*-mediated transformation, the T-DNA is randomly inserted into the host genome. With biolistic bombardment, the CRISPR reagents can be delivered as DNA, mRNA and proteins. Finally, we look at delivery of CRISPR-Cas9 as RNPs. This delivery method is preferable because the complex might exist only transiently in cells, thus minimizing potential off-target effects [19,20].

We narrowed our study selection using PRISMA guidelines [21]. Our search encompassed the past five years of research to highlight the current knowledge around genetic engineering and genome editing. We gather the potential intended and unintended changes associated with each technology from our selected studies in this article, with a special focus on the unintended changes. Unintended consequences occurring elsewhere in the host genome can produce unforeseen, adverse effects. In addition, we look at the analytical methods that were employed to identify these intended and unintended changes and take a critical look at whether these analytical methods are suitable and/or sufficient to detect these changes. With an ever-increasing toolbox at the disposal of researchers, we hope that this review can offer an insightful look at the current landscape.

## 2. Materials and Methods

### 2.1. Eligibility Criteria, Sources and Search Strategy

PRISMA guidelines adapted to our purpose were followed [21]. A systematic literature review for five chosen methods was conducted on *Agrobacterium tumefaciens*-mediated gene transfer, biolistic bombardment, and CRISPR-Cas9 delivered via *Agrobacterium*-mediated gene transfer (DNA-based), biolistic bombardment (DNA-based) and as ribonucleoprotein complexes (RNPs). The keyword search string included the name of method (including variations/derivations on the name) AND (plant OR crop) AND generic GM terms AND possible genomic outcomes. The final search strings can be found in Online Resource S1. There was a language limitation to our review, as only publications written in English were considered. Therefore, we acknowledge that this might be bias towards ‘country’ contributions. In addition, we excluded studies that were not published in peer-reviewed scientific journals.

Preliminary searches were conducted to collect relevant publications relating to the research question. A list of keywords (search terms) was compiled from this cache of articles, which formed the base of the search strings. Synonyms were added, and terms adjusted to accommodate truncations, plurals and alternative spellings. Search terms were organized into three strings related to the inclusion criteria of the study. The first string relates to genetic modification, the second to omics-related techniques, and the third to genetically modified plants (Online Resource S1) largely based on the list of approved GM plants on the ISAAA list. When the searches were performed, the strings were linked by the “AND” operator.

Each search was conducted separately and only the name of each method was altered between each search. The Web of Science Core Collection database was used, and only articles attainable through the Arctic University of Norway’s system. Our inclusion criteria involved only articles focusing on plants, only primary research articles (no reviews, no gray literature), only English language articles, and articles in the time period of 2017–June 2021 (month that search was completed). All hits were imported into EndNote software version X9. Duplicates that were found after import into EndNote were removed.

### 2.2. Study Selection and Data Collection

*Agrobacterium tumefaciens* and biolistic bombardment articles were further narrowed down at the title/abstract level to include only those articles that performed any molecular characterization of the T-DNA insertion. Articles that included *A. rhizogenes* and transient expression were excluded. This was done to focus the review on commercially available (and most relevant) transgenes.

CRISPR-Cas9 articles were further filtered using a keyword search for the specific delivery method or delivery form: *Agrobacterium*, biolistic bombardment, RNPs (Online Resource S1). Only CRISPR articles that used Cas9 were included. The names of each delivery method or delivery form also included variations on the name and can be found in Online Resource S1.

The need for inter-reviewer agreement was bypassed because only one person conducted the article screening process. If confusion or doubt existed at the title/abstract level, the potentially relevant articles were checked at full text.

A custom Google form was created to collect information from each full text paper. Several categories were created within the Google form to gather descriptive information on each paper, study metrics, as well as scientifically important and relevant questions, such as genomic outcomes and analytical methods that were employed in the study. Data was manually extracted from each full text paper and inputted into the custom Google form that was automatically formatted into a spreadsheet. The list of all articles can be found in Online Resource S2.

### 2.3. Data Handling and Evidence Synthesis

Some articles were entered into the custom Google form more than once in order to disentangle results from multiple experiments, e.g., using multiple species, using multiple plasmids, or using multiple delivery methods. In the case where articles used multiple delivery methods, separate entries were inputted, thereby altering the final count of articles for each method examined. If data was missing or not applicable, “none” or “N/A” was inputted into the Google form.

The results from genomic outcomes were grouped into categories—mutation, SNP, INDEL, rearrangement, sequence truncation, chromosomal damage, chromosome instability/imbalance, read-through, vector backbone insertion and multiple copy number inserts—for greater ease of understanding the genomic impacts (Table 1). The results for analytical methods were further divided into 11 subcategories—PCR-based, Sanger sequencing-based, Next-Gen sequencing-based, affinity-based methods (Southern blot, Western blot, ELISA, etc.), mass spectrometry (metabolites, non-affinity proteomics), spectroscopic-based, electrophoresis (agarose, capillary, etc.), microscopy, enzymatic assays (T7, GUS histochemical assay), bioinformatics (prediction, in silico analyses, sequence queries), and other (phenotyping strategies, tolerance tests, field tests, etc.) (Table 2). Detailed graphs of genomic outcomes and tables of analytical methods can be found in Online Resource S3 and S4.

### 2.4. Evidence Synthesis and Meta-Analysis

We used Microsoft Excel to synthesize data from the evidence table. Data was plotted for information regarded as main categories. For some data, it was not possible to perform a meta-analysis due to the heterogeneous nature of the study design and results.

Regarding study metrics, the number of articles per country were plotted into a map, a line graph illustrated the number of studies published per year, and a circle graph represented author affiliations. Under author affiliations, four categories were created: industry, research institute, university, and other. The category of “research institute” signifies a non-profit organization whose primary mission is research, independent of a university (e.g., national academy of sciences, national research centers). Descriptive statistics were created for species, tissue type and trait categories. A bar graph was created to illustrate the number and frequency of each species represented in the literature review, a pie chart shows the different tissue types used in experiments, and a funnel chart was plotted to display the range and frequency of traits represented in the studies included in this review. Lastly, data for genomic outcomes were grouped according to the categories in Table 1, while data for analytical methods were sorted into the categories listed in Table 2. Then, pie charts were generated for the genomic outcomes and analytical methods for each GM or gene editing method. Values in the pie charts represent the proportion of each genomic outcome or analytical method of the total number of genomic outcomes or analytical methods; respectively, for that GM or gene editing method. This systematic review follows the Preferred Reporting Items for Systematic Reviews and Meta-Analyses (PRISMA) guidelines [21].

## 3. Results

**Figure 1:**
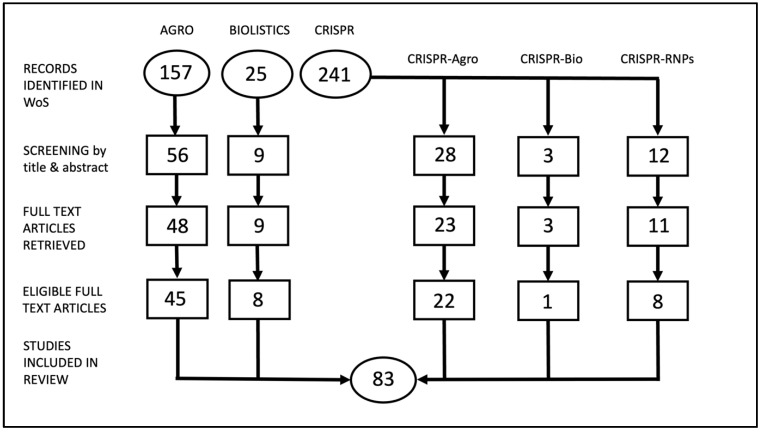
Flowchart of record retrieval and filtering used in the systematic literature review. Agro = *Agrobacterium*-mediated gene transfer. Bio = Biolistics. CRISPR = CRISPR-Cas9. RNPs = ribonucleoproteins.

We conducted a systematic literature review from the past 5 years for five genetic engineering or gene editing technologies to represent the current landscape of these technologies. After filtering steps, we narrowed down our search to our target set of articles. There was a range (2–45) of number of articles represented for each technology (Figure 1). From these articles, we focused on genomic outcomes, and especially unintended consequences, resulting from these technologies and analytical methods that were used in the articles.

*Agrobacterium*-mediated gene transfer and biolistic bombardment are two types of genetic engineering technologies that have existed for decades. As expected, biolistic bombardment is still being used for plant species that are recalcitrant to genetic transformation. Many of the articles that used *Agrobacterium*-mediated gene transfer are demonstrating proof-of-concept not just for agronomically desirable traits, but also for medical applications, such as the production of pharmaceutical proteins [22,23].

A total of 423 articles were initially retrieved from the Web of Science database according to our search criteria (Figure 1). We then filtered the articles at the title and abstract level based on further criteria, such as mention of the method that was employed in either the title or abstract, mention of any type of molecular characterization that was employed in the analyses, and only plant articles. Articles were further filtered to exclude non-relevant criteria that was not detected at the title and abstract level (transient expression, non-plant articles, protocols, delivery methods not included in this literature review, repeat articles) to yield the final set of articles that were included in the systematic literature review. A final count of 83 articles was included in our literature review.

**Figure 2:**
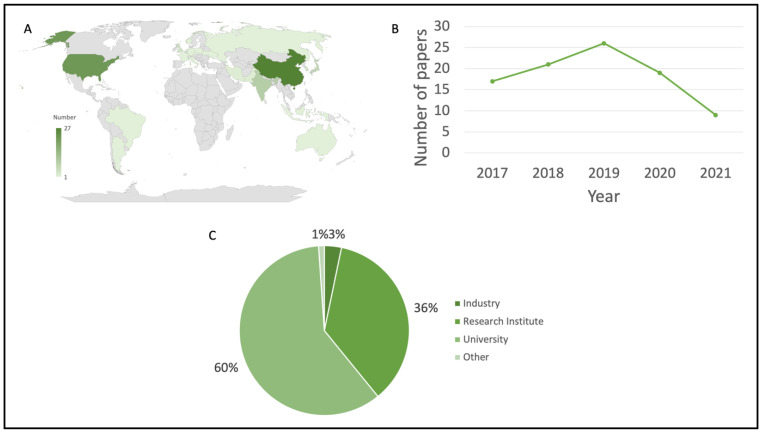
Study metrics included in the literature review. (**A**) Number of articles per country. Legend range from –127 articles. (**B**) Number of studies per year. (**C**) Author affiliation.

This literature review included articles published from 2017–2021. The majority of articles published during the five year period included in the literature review were from China (27) and the USA (22) (Figure 2A). The next highest were from India (9), Japan (7), South Korea (4) and the UK (4). A range of 17–26 articles were included in this literature review per year. Since only half of 2021 was included in the literature review, only nine articles were included. The year 2019 was a fruitful year for publishing (Figure 2B). A total of 26 articles included in the literature review were from 2019. We suspect that many more articles from 2021 would be included if the entire year was taken into account. Not surprisingly, more than half of the articles came from universities (60%) (Figure 2C). Research institutes comprised about one-third of author affiliations. Only two articles were from industry.

**Figure 3:**
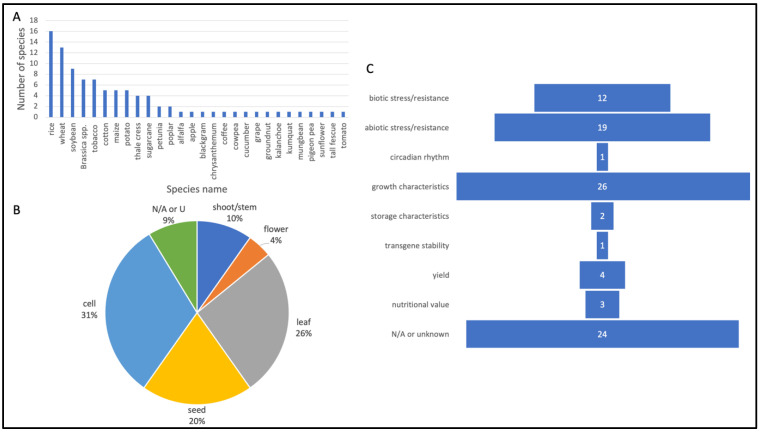
Number of publications for main study categories. (**A**) Species represented. (**B**) Tissue type used in experiments. “N/A or U” refers to “not applicable or unknown.” (**C**) Traits represented.

A wide range of plant species were represented in the literature review. Unsurprisingly, economically important crops, such as rice, wheat and soybean, comprise the top number of species that were included (Figure 3A). Many of the other articles focused on niche plant species that hold country-specific importance (e.g., kumquat, mungbean). Leaves, seeds (e.g., embryo) and cell-based material such as calli and protoplasts were the plant materials most often used for transformation and gene-editing experiments (Figure 3B). Flowers and shoots/stems were also used. Growth characteristics (approximately 28%) and abiotic stress/resistance (e.g., drought, salinity oxidative stress tolerance) (approx. 20%) were among the top desired traits that researchers used in their experiments (Figure 3C). Another notable characteristic was biotic stress/resistance (e.g., bacterial, fungal, viral, insect resistance) (13%). One paper investigated T-DNA stability and expression in field propagated sugarcane over four vegetatively propagated generations to demonstrate the commercialization potential of GM sugarcane [24]. Multiple transgene events in three commercial sugarcane varieties were generated via *Agrobacterium*-mediated transformation.

### 3.1. Genomic Outcomes

We define the intended outcomes as complete, single copy integration of T-DNA in *Agrobacterium* and biolistics, while an intended outcome in CRISPR-Cas9 is defined as a small nucleotide change at a specified target site by a single double-stranded cut made at a specified target site. Therefore, we define an unintended outcome as anything that is not an intended outcome. Such a definition is also in line with EFSA and the AHTEG guidelines for GMO risk assessment [11,12].

#### 3.1.1. Genomic Outcomes of Agrobacterium-Mediated Gene Transfer

*Agrobacterium*-mediated transformation typically involves plasmid DNA containing a gene of interest that is transferred into plant cells. The DNA cassette that harbors the gene of interest and an antibiotic selection marker is randomly integrated into the plant’s genome. A total of 45 articles were included.

**Figure 4:**
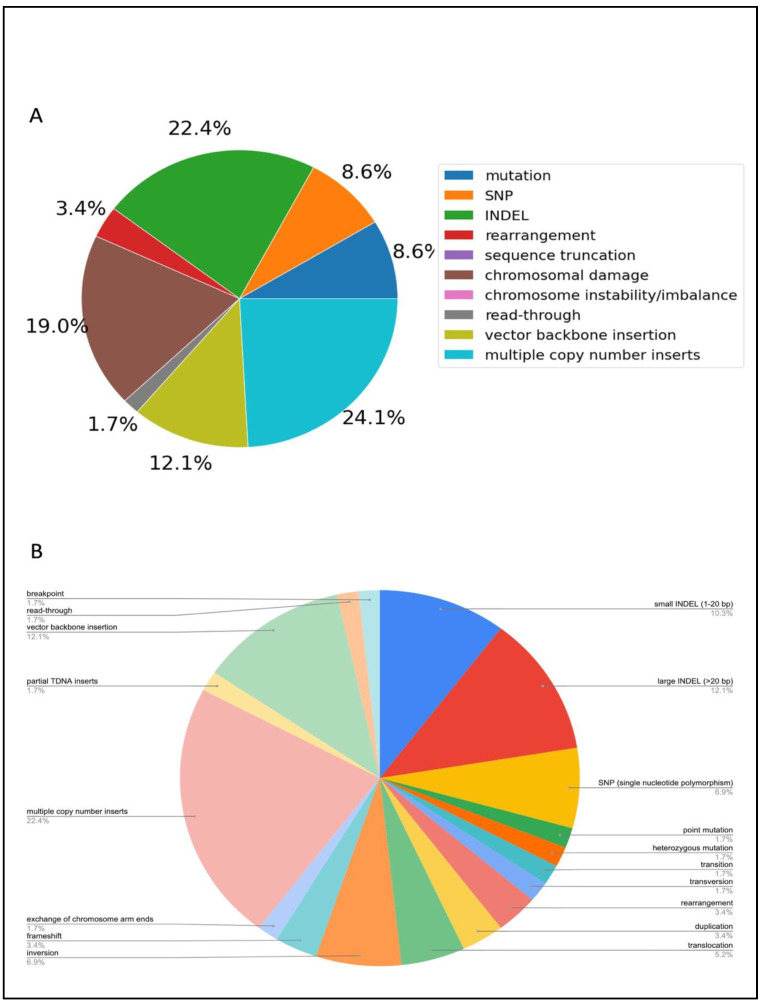
Genomic outcomes from *Agrobacterium*-mediated transformation. N = 45 articles. (**A**) Each type of genomic outcome represented as a percentage of total number of genomic outcomes. (**B**) Detailed breakdown of genomic outcomes.

The three largest genomic outcomes represented in the literature were INDELs, chromosomal damage and multiple copy number inserts (Figure 4). As the literature has previously reported, multiple copy number inserts, vector backbone insertion, and read-through resulting from *Agrobacterium* transformation are common [25]. Other unintended consequences included inversions, translocations and exchange of chromosome arm ends (Figure 4B), revealing the range and extent of genomic damage that can occur as a result of *Agrobacterium* transformation. Large deletions were also detected in many articles.

A few articles stood out from the rest. Schouten et al. [26] transformed *Arabidopsis thaliana* lines with floral dip transformation. Using Illumina and PacBio sequencing, they found deletions and translocations at T-DNA inserts. Most deletions were small in size; however, larger deletions a few thousand bp in size were also detected. A 736 kb deletion, containing 214 genes, was found in chromosome 1. This deletion contained a T-DNA insertion at the start of the deletion as well as a translocation from chromosome 3. Surprisingly, a 50-bp fragment of the gfp gene from the T-DNA insert was found more than 2 kb away from either border. The authors claimed that this is their first known report of a T-DNA fragment of this size without an accompanying T-DNA border sequence. Such large deletions and translocations were only detected because a PacBio long-run sequencing strategy was applied.

Jupe et al. [27] performed molecular characterization of *Arabidopsis thaliana* T-DNA insertion lines from the SALK, SAIL and WISC T-DNA insertion collections using different sequencing and optical mapping technologies. Their findings revealed large structural variations from 27 kb to 236 kb as a result of T-DNA insertions. The authors discovered that the T-DNA insertion was a mix of concatenated insertion fragments that could cause intra- and interchromosomal rearrangements. Multiple translocations and exchange of chromosome arm ends were detected. Though not a focus of this literature review, their results also revealed epigenomic changes at T-DNA insertion sites.

Skarzynska et al. [28] analyzed transgene insertion sites and variant prediction across three transgenic cucumber lines. They found multiple T-DNA copy insertions as well as vector backbone insertions in the analyzed lines. The T-DNA insertion sites also contained deletions in genomic DNA ranging from 95–1304 nt long. Using prediction software to assess the effect of polymorphisms that were identified in the variant analysis, the researchers determined that less than 2% of the polymorphisms identified have high impacts. Polymorphisms were induced by in vitro culturing rather than by the transformation process itself. Molecular characterization of the insertion site illustrates potential gene expression alteration.

In addition to common previously known genomic outcomes such as multiple copy inserts and vector backbone insertion, more severe genetic or genomic outcomes have been revealed with recent research, such as large genomic rearrangements, which include translocations and inversions.

#### 3.1.2. Genomic Outcomes of Biolistic Bombardment

Biolistic bombardment delivers DNA into plant cells through the high speed, high pressure bombardment of tiny beads, typically gold or tungsten, that are coated with the DNA-of-interest. The DNA is then incorporated randomly into the plant genome. A total of seven articles were included in this section.

**Figure 5:**
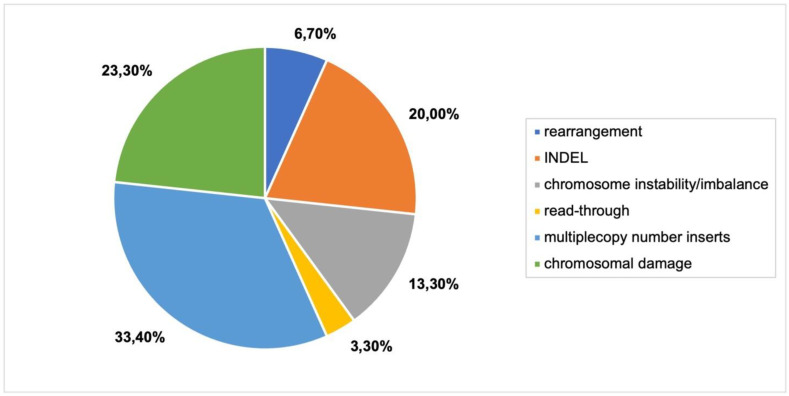
Genomic outcomes from biolistic bombardment. N = 7 articles. Colors in graph correspond to legend in Figure 4A.

Multiple copy number inserts, chromosomal damage and INDELs were the most frequently reported genomic outcomes in biolistic bombardment experiments (Figure 5). More severe genomic outcomes, such as evidence of chromothripsis and breakage-fusion-bridge cycling, were reported (Online Resource S3, Figure 1). Segmental duplications, rearrangements and copy number oscillations were also detected (Online Resource S3, Figure 1).

In particular, Liu et al. [29] performed a thorough examination of the genomic effects of biolistic bombardment. They transformed 48-kb linear lambda phage DNA into rice and maize with selectable marker plasmids. Genomic DNA from cultured callus tissue was used for sequencing analyses to reveal transformation-specific effects. The authors uncovered several instances of severe genomic damage as a result of biolistics in rice and maize, and among them were chromosome truncations, large deletions, partial trisomy, evidence of chromothripsis and breakage-fusion bridge cycling. There were several instances of large-scale arrays containing lambda and plasmid DNA mixed with genomic DNA that had been created by either nonhomologous or microhomology-mediated joining. The researchers also uncovered evidence of homology-directed repair in genomic regions that were broken and fragmented but perfectly repaired. The detailed analysis from Liu et al. [29] provides an unprecedented look at the disruptive force that biolistic bombardment can create in the plant genome.

#### 3.1.3. Genomic Outcomes of CRISPR-Cas9 Delivery via Agrobacterium

The Cas9-gRNA system can be delivered into plant cells as DNA via *Agrobacterium*-mediated gene transfer to create site-specific mutagenesis. The CRISPR-Cas9 construct may be integrated into the plant genome using this method. A total of 22 articles were included in this analysis.

**Figure 6:**
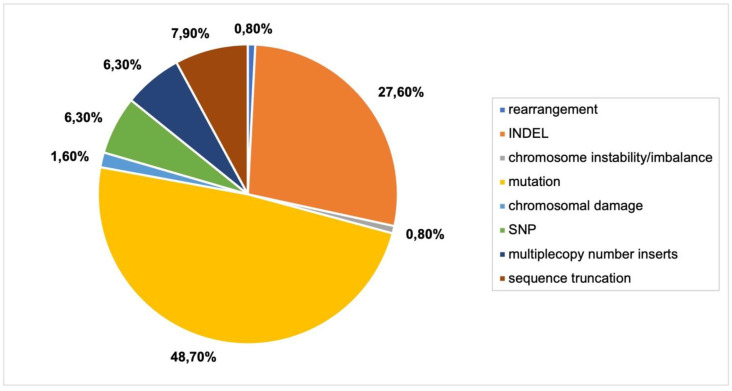
Genomic outcomes from CRISPR-Cas9 via *Agrobacterium*. N = 22 articles. Colors in graph refer to legend in Figure 4A.

Different types of mutations were the most commonly reported genomic outcomes from CRISPR-Cas9 delivery via *Agrobacterium* (Figure 6). Commonly reported mutations were homozygous, heterozygous, monoallelic, biallelic and in one case, biallelic mutations (Online Resource S3, Figure 2). Biallelic mutations refer to both alleles of a gene having different mutations in both copies of that gene. Chimeric mutations, where multiple mutations occur in the alleles of a gene, were reported. INDELs, large and small, were also frequently detected (27.6%). Some articles reported novel mutations in subsequent generations [30,31].

Char et al. [30] used a *Agrobacterium*-delivered CRISPR-Cas9 system for targeted mutagenesis in maize. They used a public sector system that can clone up to four guide RNAs for single or multiple gene targeting. They targeted two duplicated genes in two maize gene families: *Argonaute 18* (*ZmAgo18a* and *ZmAgo18b*) and *dihydroflavonol 4-reductase* or *anthocyaninless* genes (*a1* and *a4*). The duplicated genes in each family were found on two different chromosomes. The researchers designed two gRNAs to target two sites within each allele. A single plasmid Cas9-gRNA binary construct was created for transformation. For Ago18, an additional plasmid was designed to target both copies simultaneously. They reported transgenic events with mono- or diallelic mutations in a single locus and different combinations of mutations occurring in two loci in over 70% of mutants in the T_0_ generation in the two genotypes used. Furthermore, the authors reported novel site-specific mutations caused by a heritable active CRISPR-Cas9 transgene in subsequent generations. No specific off-targeting analysis was performed.

Fossi et al. [32] focused on comparing genomic instability resulting from potato regeneration from either protoplasts or stem explants. Plants from protoplasts were first cultured axenically then regenerated for a total of 15 regenerants. These regenerants were compared to 33 transformed regenerants from stem internodes in *Agrobacterium* co-cultivation experiments. A set of 8 plants propagated from cuttings served as a control. In the *Agrobacterium* experiments, two vectors were used–one CRISPR-Cas9 and one not, but it was unclear which vector was used in which individual co-cultivation experiment. Regenerants from protoplasts displayed genome instability in the form of terminal arm changes, pentasomy, and highly fragmented chromosomal regions. In the *Agrobacterium* co-cultivation experiments, the potato regenerants from stem explants displayed less severe genomic damage than potato protoplast regenerants; however, trisomy and large-scale deletions were still detected. The authors reported instability patterns in certain genomic areas that suggest these sites to be more susceptible than others. A detailed examination of the target site was not described in the paper, and therefore no off-targeting analysis was conducted.

CRISPR-Cas9 delivered via *Agrobacterium* is an efficient method of gene-editing, but the design and optimization must be carefully considered for accuracy. The ability to use one gRNA to target more than one site is a plus for researchers working with polyploids. The continued action of a heritable Cas9 may or may not be problematic.

#### 3.1.4. Genomic Outcomes of CRISPR-Cas9 via Biolistics

CRISPR-Cas9 can also be delivered as DNA via biolistic bombardment, resulting in possible integration of the CRISPR-Cas9 construct into the plant genome. Only two articles were included after filtering.

**Figure 7:**
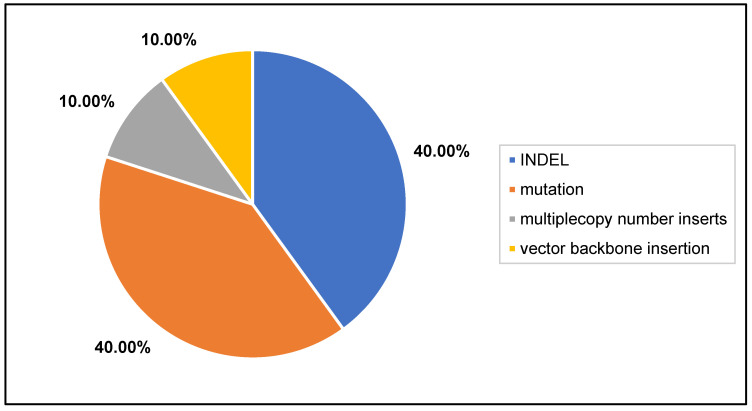
Genomic outcomes from CRISPR-Cas9 via biolistic bombardment. N = 2 articles. Colors in graph correspond to legend in Figure 4A.

Mutations and INDELs were the majority of the genomic outcomes from the two articles that were included in this section (Figure 7; Online Resource S3, Figure 3). Multiple T-DNA insertions and vector backbone inserts were also detected, not surprisingly, as biolistic bombardment often results in these unintended consequences.

Adachi et al. [33] edited soybean embryos to lack *Gly m Bd 30K* gene, which is an allergenic gene. Embryos were bombarded with the p30K-hyg expression vector and maintained in liquid media containing hygromycin. Five transgenic lines were selected 3 months post-bombardment. Two transgenic embryo lines displayed insertions of 600 and 133 nucleotide vector backbone sequence at the target locus. Deletions also predominated. The mutations at the target locus were found to all contain biallelic mutations. An off-target analysis found no evidence of mutations at three potential sites containing 4-nucleotide mismatches to the gRNA. Out of five transgenic embryo lines, two lines were regenerated into plantlets. Segregation analysis of transgene integration in the T_1_ progenies indicated that these two lines most likely contained a single copy of the transgene or a single locus containing more than one copy. Therefore, the researchers speculated that failure of regeneration from the other three lines could be attributed to integration of many transgene copies. Analysis of DNA extracted from cotyledons of T_1_ seeds from these two regenerated lines confirmed transmission of the mutations at the target locus in the T_1_ generation. The T_2_ generation was generated from Cas9-free plants containing homozygous mutant alleles. SDS-PAGE and immunoblot analyses confirmed the lack of Gly m Bd 30K protein accumulation in all four mutant genotypes. Total RNA was extracted from mature T_3_ seeds and the absence of expression of *Gly m Bd 30K* gene was confirmed in three mutant genotypes.

As described in more detail below, Banakar et al. [34] detected fragments of plasmid and chromosomal DNA resulting from biolistic bombardment experiments with DNA.

Based on this literature review, CRISPR-Cas9 delivered as DNA via biolistic bombardment is not a commonly used delivery method. Adachi et al. [33] were able to confirm heritability of mutations in subsequent generations and the absence of protein accumulation and gene expression of the target.

#### 3.1.5. Genomic Outcomes of CRISPR-Cas9 as RNPs

The Cas9-gRNA can be delivered into protoplasts or plant tissue as a protein/RNA complex, also known as a ribonucleoprotein or RNP. Delivering CRISPR-Cas9 as RNPs into plant cells eliminates transgene integration and may lower the risk of unwanted genetic changes. Studies used PEG-mediated transfection, lipofection reagents, or biolistic bombardment to introduce the RNP complex into the cell. Eight articles were included.

**Figure 8:**
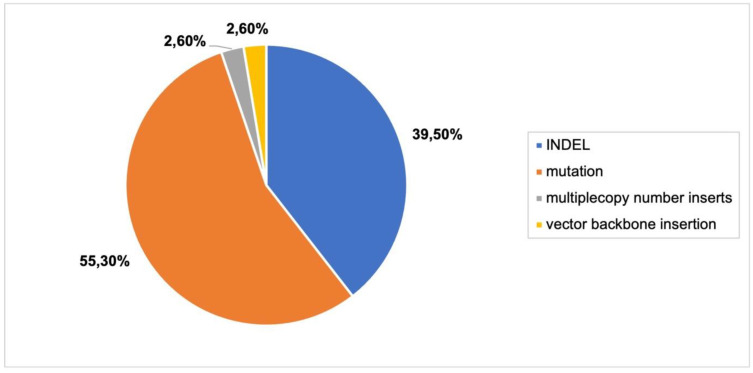
Genomic outcomes from CRISPR-Cas9 as RNPs. N = 8 articles. Colors in graph correspond to legend in Figure 4A.

Different types of mutations and INDELs were an overwhelming majority of the reported genomic outcomes (Figure 8; Online Resource S3, Figure 4). Similar to delivery via *Agrobacterium* and biolistic bombardment, articles reported homozygous, heterozygous, monoallelic, biallelic and chimeric mutations. One paper found a positive correlation between exposure time and mutagenesis frequency [35].

Initial experiments conducted in Liang et al. [36] involved genome editing on bread wheat protoplasts using ribonucleoproteins (RNPs) by PEG-mediated transfection. Cas9 was expressed from *E. coli*, purified and mixed with in vitro transcribed sgRNA targeting the *TaGW2* gene controlling grain weight. The sgRNA simultaneously targeted the three homoeologs in bread wheat, with only a single nucleotide mismatch in the A subgenome’s target site, which was designated as an off-target site. DNA from protoplasts was extracted 48 h post-transfection. The CRISPR-Cas9 RNP complexes were highly active with on-target mutation frequencies of 21.8% (target in the D subgenome) and 33.4% (target in the B subgenome). The frequency of off-targeting in the A subgenome was 5.7%. Next, the RNPs were tested in immature wheat embryo cells using biolistic bombardment. DNA was extracted from pooled immature embryos 2 days post-bombardment and analyzed for mutations at the cleavage sites using Illumina targeted deep sequencing. Mutagenesis frequencies at *TaGW2-B1* and *TaGW2-D1* were 0.18% and 0.21%, respectively, while the off-targeting frequency at *TaGW2-A1* was 0.03%. Then, the bombarded embryos were regenerated and screened to detect mutations in the targets. Out of 28 *tagw2* mutants from 640 bombarded immature embryos, half contained INDELs in *TaGW2-B1*, all had INDELs in *TaGW2-D1*, and no mutations were found in *TaGW2-A1*. An additional 20 off-target sites ranging from 2–5 nucleotide mismatches were investigated in the regenerated mutants. No off-targeting was found in the edited plants using either PCR-RE assay or Sanger sequencing of PCR products. In parallel experiments, gw2-sgRNA and Cas9 were expressed from a plasmid (pGE-TaGW2). In protoplast transfection experiments, on-target mutagenesis frequencies were similar to RNPs while they were five-fold higher in bombardment experiments using plasmid compared to RNPs. Off-target mutagenesis frequencies in both protoplast and immature embryo experiments were higher with pGE-TaGW2 than with gw2-RNPs.

Murovec et al. [37] genome-edited three *Brassica* species—*B. oleracea*, *B. napus* and *B. rapa*—using preassembled ribonucleoprotein complexes (RNPs) from purified recombinant Cas9 from *E. coli* and sgRNAs that were either in vitro-transcribed or synthesized. RNPs were delivered into protoplasts using PEG 4000. A total of four sgRNAs were tested-two targeting the FRIGIDA gene and another two targeting the phytoene desaturase gene. Varying amounts of sgRNA and Cas9 were also tested: 7.5, 15, 30 and 60 ug. RNP complexes were prepared by mixing and incubating purified Cas9 with in vitro-transcribed sgRNA, then adding an equal volume of 40% PEG 4000. DNA from protoplasts was extracted at either 24 or 72 h post-transfection. Mutation frequency results were calculated as the INDEL percentage detected at the target site. *B. napus* protoplasts did not display any detectable mutations after transfection. Mutation frequencies of 0.09 to 2.25% were obtained in *B. oleracea* and 1.15 to 24.51% in *B. rapa*, and depended on the locus that was targeted as well as the RNP amount used. The mutation frequency did not increase from 24 to 72 h post-transfection. A more detailed sequence analysis performed on *B. rapa* cells post-transfection using the highest amount of Cas9 with sgRNA (60 μg each) revealed that most INDELs surrounding the target sites were 1 bp long and 1–4 nucleotides around the cleavage site. An off-targeting analysis was not performed in this study.

Another important paper to highlight came from Banakar et al. [34]. Three delivery platforms were compared: RNPs co-delivered with plasmid DNA containing a selectable marker gene via biolistic bombardment, plasmid DNA via biolistic bombardment, and plasmid DNA via *Agrobacterium*-mediated transformation. In the CRISPR-Cas9 RNPs co-bombarded with plasmid DNA containing a selectable marker gene experiments, two guide RNAs were designed to target the same rice phytoene desaturase (*OsPDS1*) gene 95 nucleotides apart. CRISPR reagents were purchased commercially. The RNP complex was formed and mixed with plasmid DNA carrying a hygromycin B resistance gene and gold particles, then bombarded into rice embryos. The biolistic bombardment experiments were executed in the same manner excluding the RNP complex. Both types of experiments then screened on selection media and regenerated plants. *Agrobacterium* experiments used the same constructs that were used in biolistic bombardment experiments. On-target mutations were generated from all delivery methods. The authors reported frequent random DNA fragments originating from plasmid and chromosomal DNA inserted at target sites in both biolistic bombardment approaches at around the same frequency. In addition to small INDELs, large on-target insertions were comprised of a plasmid DNA vector backbone sequence, T-DNA of the vector and/or the PDS1 gene. In *Agrobacterium* experiments, no evidence of random DNA insertions was found. No off-target mutations were found in two off-target sites that were analyzed in seven mutant lines from all three delivery methods.

RNPs may be a preferred method of delivering CRISPR-Cas9 into plant cells that bypasses transgene integration and produces relatively lower unwanted genetic changes. Since regeneration from protoplasts is difficult in major cereal crops, more research will need to be done to create an efficient protocol for CRISPR-Cas9 RNP delivery in these economically important crops.

### 3.2. Analytical Methods Used

**Figure 9:**
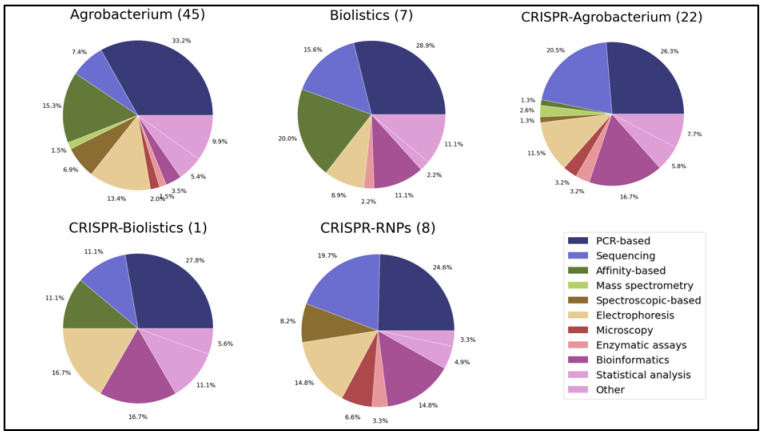
Analytical methods applied in the reviewed papers for the study of genetic outcomes of genetic engineering. Number of articles included in each method is represented within brackets next to the title of each pie chart. Each method is represented as a percentage of total number of methods. The “Other” category includes methods such as staining, field experiments, stress tests, etc.

Next, we focused on the analytical methods for the identification of potential intended and unintended changes. Individual analytical methods were organized into 10 categories represented in Figure 9. Individual breakdowns can be found in Online Resource S4.

#### 3.2.1. Analytical Methods Used in the Characterization of Agrobacterium-Mediated Gene Transfer-Induced Alterations

The most commonly used analytical methods in *Agrobacterium* transformation experiments included PCR-based assays (33%), and affinity-based assays (15%) (Figure 9). Many of the articles reported using PCR and Southern blotting for molecular characterization of the T-DNA insertion (Online Resource S4). qPCR, qRT-PCR, and RT-PCR were also employed. Naturally, agarose gel electrophoresis following PCR was often used also. A few articles used Illumina targeted deep-sequencing in their analyses. This method allows for the identification of rare variants by sequencing specific areas of interest to a very high depth.

Schouten et al. [26] resequenced transgenic *Arabidopsis thaliana* lines using both Illumina (Illumina Hi Seq 2000, 100-nt paired-end sequencing) and PacBio (RS-II) sequencing technologies. When putative translocations flanking T-DNA inserts were detected, the Illumina paired-end reads were insufficient to elucidate the resulting structural variation so the researchers had to supplement the data with PacBio sequencing to confirm the putative translocations. As there were multiple T-DNA inserts in each plant, it was necessary to use the PacBio data to assist in the molecular characterization of entire T-DNA inserts.

Jupe et al. [27] used Oxford Nanopore Technologies sequencing and Bionano Genomics optical mapping to elucidate complex structures in the genome as a result of T-DNA insertions. The long-range sequencing platforms enabled the detection of previously unknown changes in the genome.

In Skarzynska et al. [28], transgenic cucumber lines were resequenced using 126.6–126.8 million paired-end Illumina reads per plant to analyze the T-DNA insertion site and other genomic changes. They then performed variant prediction and annotated the variants to predict their probable effect on genes and proteins.

#### 3.2.2. Analytical Methods Used in the Characterization of Biolistic Bombardment-Induced Alterations

The most commonly used analytical methods in biolistic bombardment experiments included PCR-based assays (29%) and affinity-based assays (20%) (Figure 9). PCR, RT-PCR, and qRT-PCR were used often (Online Resource S4). Western and Southern blotting were commonly used.

Liu et al. [29] achieved an extremely high level of detail in their paper by utilizing a combination of short-read Illumina and long-read PacBio sequencing as well as Bionano optical mapping in their analyses. This enabled them to precisely reconstruct large genomic areas where complex rearrangements composed of introduced DNA interspersed with genomic DNA fragments occurred. In particular, Bionano optical mapping aided the analysis in identifying large structural variation in addition to insertions < 100 kb in size. PacBio sequencing was instrumental in the analyses to discern a novel sequence insertion greater than 1.6 Mb that was revealed to contain 1810 lambda fragments from 31–11,387 bp in size.

#### 3.2.3. Analytical Methods Used in the Characterization of CRISPR-Cas9 Delivery via Agrobacterium-Induced Alterations

The most commonly used methods included PCR-based assays (26%), sequencing (21%), and bioinformatics (17%) (Figure 9). PCR, RT-PCR, qRT-PCR, RT-qPCR were often used (Online Resource S4). Sanger sequencing and agarose gel electrophoresis are still being used. Illumina targeted deep sequencing, a PCR-based sequencing approach, was used to analyze the specific mutations in a few studies. Bioinformatic techniques were commonly used to analyze on- and off-target sites.

Fossi et al. [32] used whole genome sequencing to assess copy number changes. Each plant was sequenced with an average of 7.58 million Illumina reads. Libraries from protoplasts were sequenced on an Illumina HiSeq 4000 in 100-nt single-end mode, while those from explants were sequenced on an Illumina NovaSeq 6000 in 150-nt paired-end mode. Sequencing reads were binned into non-overlapping bins using sequencing counts from an individual wild-type control plant for comparison of deviations from the expected four copies. This dosage analysis was used to determine copy number variation across chromosomes.

#### 3.2.4. Analytical Methods Used in the Characterization of CRISPR-Cas9 via Biolistics-Induced Alterations

PCR-based assays (28%), bioinformatics (17%), and electrophoresis (17%) were the most commonly used methods (Figure 9). Agarose gel electrophoresis and Sanger sequencing were also popular.

Adachi et al. [33] employed CRISPR-P to select the gRNA sequence. They also used PCR, semi-quantitative RT-PCR, and immunoblot analysis to confirm the absence, expression and accumulation of their target gene. PCR and cleaved amplified polymorphic sequence analysis was also used for genotyping. Sanger sequencing was used for analysis.

#### 3.2.5. Analytical Methods Used in the Characterization of CRISPR-Cas9 as Rnps-Induced Alterations

The majority of analytical methods used were PCR-based assays (25%) and sequencing (20%). PCRs to carry out in vitro digestion assays were common. Sanger sequencing and Illumina targeted deep sequencing were popular sequencing methods used. Illumina targeted deep sequencing was used in multiple studies. Bioinformatics software was also frequently used to analyze on- and off-targeting.

Liang et al. [36] performed PCR-RE assay and Sanger sequencing to identify mutations in regenerated plants. Illumina targeted deep sequencing was employed to detect mutations as defined by INDELs at the target site.

Murovec et al. [37] used Illumina targeted deep sequencing to calculate the INDEL percentage at the cleavage site. Briefly, PCR is carried out to amplify the target site. PCR products were sequenced using the Illumina platform and possible mutations around the PAM site was detected with CRISPR RGEN Tools Cas-Analyzer and CRISPResso software.

Banakar et al. [34] used PCR and Sanger sequencing to genotype transgenic rice plants. Transgene copy number analysis was performed using PCR, Sanger sequencing and qPCR. Researchers used CGAT software to analyze off-targets, followed by PCR and Sanger sequencing.

## 4. Discussion

This literature review focused on five current genetic modification or gene/editing methods and their genomic outcomes. We then probed the literature to ascertain the methods that were employed to analyze these outcomes. The large range in the number of articles included for each technology is a consequence of our filtering procedure, but largely due to either the current attractiveness of the technology or how well-established the technology is.

### 4.1. Identification of Genomic Outcomes

Overall, most CRISPR-Cas9 publications are still mostly proof-of-concept, while the *Agrobacterium* technology is more mature. Some *Agrobacterium* articles re-examined previously regenerated transgenic lines to more clearly characterize the T-DNA insertion and unintended outcomes [27,28]. As expected, biolistic bombardment is generally used with species that are recalcitrant to *Agrobacterium* transformation. In classical GM methods, integration of vector backbone and multiple T-DNA inserts are outcomes that were previously reported and confirmed again through the recent articles. Severe genomic damage in the form of genome shattering and breakage–fusion bridge cycle were reported for biolistic bombardment.

Next, we examined gene editing technologies with a focus on CRISPR-Cas9. Although young compared to *Agrobacterium*-mediated transformation and biolistic bombardment, it is a fast-growing area of research. For CRISPR-Cas9 experiments, on-target INDELs and multiple mutation types were frequently reported, as expected. Since many articles focused solely on intended consequences, it is possible that unintended consequences and/or off-targeting go undetected and therefore have been underreported.

The ability to simultaneously target multiple cleavage sites using a single CRISPR-Cas9 complex is especially useful and efficient for researchers working with polyploids, where it would be desirable to simultaneously gene edit the target in all subgenomes. It is theorized that differential editing may be attributed to GC content, secondary structure, euchromatic vs. heterochromatic regions, etc. In a study on genome-edited bread wheat, in which protoplasts and immature embryo cells were gene edited using a sgRNA targeting all three subgenomes, it was observed that the on-target (in B and D subgenomes) versus off-target (in A subgenome) mutagenesis frequency was much higher using RNPs than with a plasmid [36]. Additionally, no mutations were found in the off-target in regenerated mutants, suggesting that CRISPR-Cas9 editing using RNPs is more specific than expressing Cas9 and sgRNA from a plasmid. The studies in this review suggest that this could be due to the transient nature of RNPs, the lack of transgene integration, and minimal exposure time of the CRISPR-Cas9 complex to the genome. On the other hand, new evidence in zebrafish shows that structural variants can occur at on- and off-target sites via CRISPR-Cas9 editing through RNPs, providing further evidence that this technology may not be as specific as previously thought [38].

CRISPR-Cas9 delivered through biolistics as DNA or RNPs was not found to be a common delivery method during the timeframe encompassed in this literature review. CRISPR-Cas9 as RNPs has been touted as a promising alternative to using a plasmid for avoiding foreign DNA integration and could lower the risk of unintended genetic consequences. In the case of Liang et al. [36], where the off-target site in one subgenome had a 1 bp mismatch to the gRNA compared to the two other subgenomes that had a perfect match, the off-targeting activity was much lower than on-target in wheat protoplasts, although not completely absent. The other articles did not perform off-target analyses. More research will need to be performed in plants to test whether the technology is as promising as expected.

Using classical GM methods to deliver CRISPR-Cas9 is still associated with the unintended outcomes that are specific to the classical GM method. As we have seen in these recent articles, using *Agrobacterium* or biolistic bombardment to deliver CRISPR-Cas9 into the cell still introduces unintended consequences such as multiple copy number inserts into the genome (Figure 6 and Figure 7), which suggests that it is not enough to merely investigate cleavage and potential off-target sites if one wishes to understand the full impact on the genome and subsequent gene expression, proteome, etc.

Depending on one’s goal, the novel mutations found in progeny as a result of a heritable active CRISPR-Cas9 complex can be either desirable or not. As Char et al. [30] suggest, this could be employed to introduce mutagenesis into species that are recalcitrant to transformation through crossing or be used in combination with the introduction of new gRNAs to create different gene edits in an already established transformant. If, however, novel mutations in progeny are undesirable from a biosafety prospective, the continuing action of Cas9-gRNA would be flagged.

### 4.2. Biased and Unbiased Analytical Methods and Their Suitability for Molecular Characterization

Many of the articles in *A. tumefaciens* and biolistic bombardment experiments use PCR and Southern blotting for molecular characterization of the T-DNA insertion. However, short PCR products that are limited to amplifying in and around the target region may not suffice in correctly characterizing the insertion event. Luckily, the trend seems to be moving away from the cumbersome Southern blotting technique for identifying transgene copy number. This method can be misleading because it is operation-dependent. RT-qPCR is a better method because of its high specificity, sensitivity and accuracy. PCR, sequencing, electrophoresis and bioinformatics play large role for CRISPR-Cas9 experiments.

The analytical methods used in the reported studies can be divided into two categories—biased and unbiased methods. The majority of analytical methods used by researchers in this literature review were biased methods. This could largely be attributed to the technologies that have been available to researchers over the past decades. However, unbiased methods can be more powerful in that they can provide a more thorough molecular characterization of the transgenic or gene-editing event. Their seldom usage thus far for these purposes could be attributed to the high price tag of unbiased sequencing services, limited resources, etc.

The question remains of whether the analytical methods are suitable for finding genomic outcomes. The lack of reporting does not necessarily indicate the absence of the outcome, and by using only biased methods, the full extent of genomic outcomes from a transgenic or gene-editing event is underestimated. This review has demonstrated that for classical GM methods such as *Agrobacterium*-mediated transformation and biolistic bombardment, next generation sequencing methods and optical mapping revealed some previously undetected unintended consequences and highlighted the severity of previously reported outcomes.

Included in our literature review were only five articles that used whole genome sequencing in their analysis methods—Schouten et al. [26] (*Agrobacterium*), Jupe et al. [27] (*Agrobacterium*), Skarzynska et al. [28] (*Agrobacterium*), Liu et al. [29] (Biolistics), and Fossi et al. [32] (CRISPR-Cas9-Agro). The use of short- and long-read sequencing technologies has revealed valuable information about genomic consequences as a result of genetic modifications and edits. Chromothripsis-like outcomes and breakage–fusion bridge cycling following biolistic bombardment [29] were two of the more severe genomic disruptions that were elucidated using whole genome sequencing. The detection of large structural variations in Jupe et al. [27] would not be possible without long-read sequencing. The copy number oscillation detected in certain chromosomal regions was facilitated by Illumina short-read sequencing technologies in Fossi et al. [32] and Liu et al. [29]. When comparing newer analytical methods in the molecular characterization of T-DNA inserts to older methods, Schouten et al. [26] pointed out that NGS can be more sensitive than Southern blotting. The short partial T-DNA fragment, which was discovered with NGS in their study, may have been missed by Southern blotting and PCR. In addition, NGS can be employed to analyze the flanking DNA around T-DNA inserts.

As new technologies are constantly evolving, a more thorough examination of prospective analytical methods should be conducted in the future. This will provide regulators working in the field of genetically modified and gene-edited organisms with valuable information on the ability to detect and identify human interventions.

### 4.3. Implications for Risk Assessment and Safety Policies

The potential for unintended genetic changes in genetically modified organisms, including gene-edited organisms, is a legitimate biosafety concern. In the current EFSA guidance for risk assessment of food and feed from genetically modified plants, unintended effects are considered differences between the GM plant and its comparator, which go beyond the intended effects of the genetic modification and could be linked to genetic rearrangements or metabolic perturbations [11]. A similar idea is also reflected in the guidance on risk assessment of living modified organisms written by the Ad Hoc Technical Expert Group (AHTEG) on Risk Assessment and Risk Management serving the United Nations Convention on Biological Diversity [12]. In the AHTEG guidance, the investigation of unintended changes in the GMO is part of the first step of conducting a risk assessment: Step 1: “An identification of any novel genotypic and phenotypic characteristics associated with the living modified organism that may have adverse effects on biological diversity in the likely potential receiving environment, taking also into account risks to human health”.

As analytical methods evolve, it is relevant to search in the scientific literature for investigations and studies on intended and unintended effects from genetic modification techniques. In addition, there is also the emergence of new technique for genomic modification, named gene-editing. The study of the impact of gene-editing in the genome of plants is still in its infancy and requires constant literature monitoring for its understanding. Such new methods can help risk assessors in providing the necessary information on the safety of these products, as well as for updating current guidelines.

## 5. Conclusions, and Future Directions

This systematic review revealed a clear lack of detailed information on experimental designs in the publications examined, which posed a limitation to our meta-analysis. In the context of unintended DNA alterations, we found a range of different sequence rearrangements ranging from the introduction of small INDELs to long sequence chromosomal duplications. The literature shows that the unintended outcomes are directly correlated to the type of analytical method used to investigate DNA sequence alterations and that most papers might have an underestimation of these effects due to lack of dedicated testing.

We found no guidelines or methodological trends regarding experimental design, choice of analytical method and statistical analysis. Therefore, for efficient regulatory implementation of such testing, there is a need to develop frameworks related to proper reporting and dedicated experimental setups. Most importantly, there is a need for a framework for the definition of biological relevance of the generated data. We have observed the same pattern when we investigated alterations in the proteomic and metabolomic profile analyses of GM plants [39]. Nevertheless, the lack of harmonized methods seems to be due to the rapid progress of omics technologies rather than inconsistent reporting. This can be observed even from the short window of five years-old papers analyzed in this study.

In summary, we conclude that new genomic techniques, such as sequencing techniques, are suitable tools to comprehensively screen for alterations in genetically modified plants due to their high throughput and untargeted nature. In light of the speed of development of new GMOs, new tools such as third generation sequencing are needed to enable a comprehensive risk assessment.

## Figures and Tables

**Table 1:** Description of genomic variations.

Name of Variation	Definition/Explanation/Comment
***Type of Variant—Nucleotide Polymorphism***	
SNP (single nucleotide polymorphism)	Nucleotide change at a single position. Point mutations, nucleotide substitutions.
InDel (Insertion/deletion)	Insertion or deletion of DNA, typically short (1–20 bp but can also be longer).
Mutation	Transitions, transversions. Heterozygous, homozygous, biallelic, and chimeric.
***Type of Variant—Structural Variants***	
Multiple copy number inserts	Describes number of T-DNA insertions into the host genome in *Agrobacterium*-mediated transformation and biolistics. Can include partial T-DNA insertions.
Vector backbone insertion	Occurrence of vector backbone inclusion in T-DNA transfer during *Agrobacterium*-mediated transformation.
Read-through	In *Agrobacterium*-mediated transformation, regions outside the T-DNA are transferred. Can be left border read-through.
Chromosomal damage	Segmental duplication, chromosome truncation.
Chromosome instability-imbalance	Trisomy, chromothripsis, breakage fusion bridge.

**Table 2:** Description of analytical methods.

Name of Method	Definition/Explanation/Comment
PCR-based	Polymerase chain reaction (PCR) used to make millions of copies of specific DNA segment. Size restrictions. Length of DNA amplified determined by polymerase used. Usually used for amplifying relatively short DNA fragments.
Sanger sequencing-based	Method for ascertaining DNA nucleotide sequence. Can sequence up to 1000 bases.
Next-Gen sequencing-based	Methods for determining nucleotide sequence of DNA or RNA. High throughput. Can determine sequence of entire genomes. Also included in this category are optical mapping technologies, spatial genomics.
Affinity-based	Methods using binding and interactions between molecules/compounds for separation and purification purposes.
Mass spectroscopy	Methods used to ascertain molecular weights and chemical structures of compounds.
Spectroscopic-based	Methods using absorption and emission of light.
Electrophoresis	Methods to separate DNA, RNA or proteins according to size and electrical charge.
Microscopy	Methods using microscopes to examine objects too small to be seen with the naked eye.
Enzymatic assays	Methods for testing activity of enzymes.
Bioinformatics	Methods for analyzing and interpreting biological data.
Other	For the sake of this paper, methods that did not fall into the previous categories.

## Data Availability

The data presented in this study are available in Supplementary Materials Online Resource S1–S4.

## Supplementary Materials

The following supporting information can be downloaded at: https://www.mdpi.com/article/10.3390/plants11212997/s1, Online Resource S1: Keywords used for literature search; Online Resource S2: List of articles; Online Resource S3: Genomic Outcomes; Online Resource S4: Analytical methods
