# Supplementary material for: Unintended Genomic Outcomes in Current and Next Generation GM Techniques: A Systematic Review
Source: Plants (Basel). 2022 Nov 7;11(21):2997. doi: 10.3390/plants11212997 (PMC9655061; doi:10.3390/plants11212997)
Supplement: Supplementary file 1 [file plants-11-02997-s001.zip › Online Resource S1 - Keywords used for literature search.pdf]

Supplementary Information

Section 1. Keywords used for literature search

Agrobacterium

(((Agrobacteri* OR tumefaciens OR "agrobacterium tumefaciens" OR "agrobacterium mediated gene transfer" OR "agrobacterium-mediated gene transfer") NOT (rhizogen* OR transient) AND (plant OR crop) AND (genetic transformation OR genetic engineering OR transgene integration OR transgene OR transgenic OR genome engineering OR gene-editing OR genome editing OR programmable nuclease OR sequence-specific nuclease OR site-directed nuclease OR SDN OR direct DNA transfer OR GMO OR genetically modified organism) AND ("vector backbone insertion" OR disruption OR rearrange* OR inversion OR translocation OR "event quality" OR "event frequency" OR read-through OR "read through" OR breakpoint OR damage OR trisomy OR "breakage-fusion bridge cycling" OR "breakage-fusion-bridge cycling" OR "breakage-fusion-bridge cycle"OR "chromosome breakage" OR shatter* OR displace* OR reassembl* OR translocat* OR duplicat* OR "broken ends" OR chromothripsis OR "transgene copy number" OR "sequence breakage" OR "illegitimate recombination" OR "molecular characterization" OR Off-target* OR "off target*" OR "unintended modification*" OR "unintended effect*")))

Biolistics

((Biolistic* OR biolistic bombardment OR "gene gun" OR "particle bombardment" OR "microprojectile*" OR "microprojectile bombardment") AND (plant OR crop) AND (genetic transformation OR genetic engineering OR transgene integration OR transgene OR transgenic OR genome engineering OR gene-editing OR genome editing OR programmable nuclease OR sequence-specific nuclease OR site-directed nuclease OR SDN OR direct DNA transfer OR GMO OR genetically modified organism) AND ("vector backbone insertion" OR disruption OR rearrange* OR inversion OR translocation OR "event quality" OR "event frequency" OR read-through OR "read through" OR breakpoint OR damage OR trisomy OR "breakage-fusion bridge cycling" OR "breakage-fusion-bridge cycling" OR "breakage-fusion-bridge cycle"OR "chromosome breakage" OR shatter* OR displace* OR reassembl* OR translocat* OR duplicat* OR "broken ends" OR chromothripsis OR "transgene copy number" OR "sequence breakage" OR "illegitimate recombination" OR "molecular characterization" OR Off-target* OR "off target*" OR "unintended modification*" OR "unintended effect*"))

CRISPR-Cas9

((((CRISPR* OR CRISPR/Cas* OR CRISPR-Cas* OR CRISPR cas OR "Clustered, regularly interspaced, short palindromic repeats") AND (plant OR crop) AND (genetic transformation OR genetic engineering OR transgene integration OR transgene OR transgenic OR genome engineering OR gene-editing OR genome

editing OR programmable nuclease OR sequence-specific nuclease OR site-directed nuclease OR SDN OR direct DNA transfer OR GMO OR genetically modified organism) AND ("vector backbone insertion" OR disruption OR rearrange* OR inversion OR translocation OR "event quality" OR "event frequency" OR read-through OR "read through" OR breakpoint OR damage OR trisomy OR "breakage-fusion bridge cycling" OR "breakage-fusion-bridge cycling" OR "breakage-fusion-bridge cycle"OR "chromosome breakage" OR shatter* OR displace* OR reassembl* OR translocat* OR duplicat* OR "broken ends" OR chromothripsis OR "transgene copy number" OR "sequence breakage" OR "illegitimate recombination" OR "molecular characterization" OR Off-target* OR "off target*" OR "unintended modification*" OR "unintended effect*"))))

CRISPR-Agro
Search within CRISPR-Cas9 results for (Agrobacteri* OR tumefaciens OR "agrobacterium tumefaciens" OR "agrobacterium mediated gene transfer" OR "agrobacterium-mediated gene transfer") in EndNote “any field”

CRISPR-Biolistics
Search within CRISPR-Cas9 results for (Biolistic* OR biolistic bombardment OR "gene gun" OR "particle bombardment" OR "microprojectile*" OR "microprojectile bombardment") in EndNote “any field”

CRISPR-RNPs
Search within CRISPR-Cas9 results for (RNP or ribonucleoprotein or rnps) in EndNote “any field”
