# Supplementary material for: Unintended Genomic Outcomes in Current and Next Generation GM Techniques: A Systematic Review
Source: Plants (Basel). 2022 Nov 7;11(21):2997. doi: 10.3390/plants11212997 (PMC9655061; doi:10.3390/plants11212997)
Supplement: Supplementary file 1 [file plants-11-02997-s001.zip › Online Resource S3 - Genomic Outcomes.pdf]

Online Resource 3

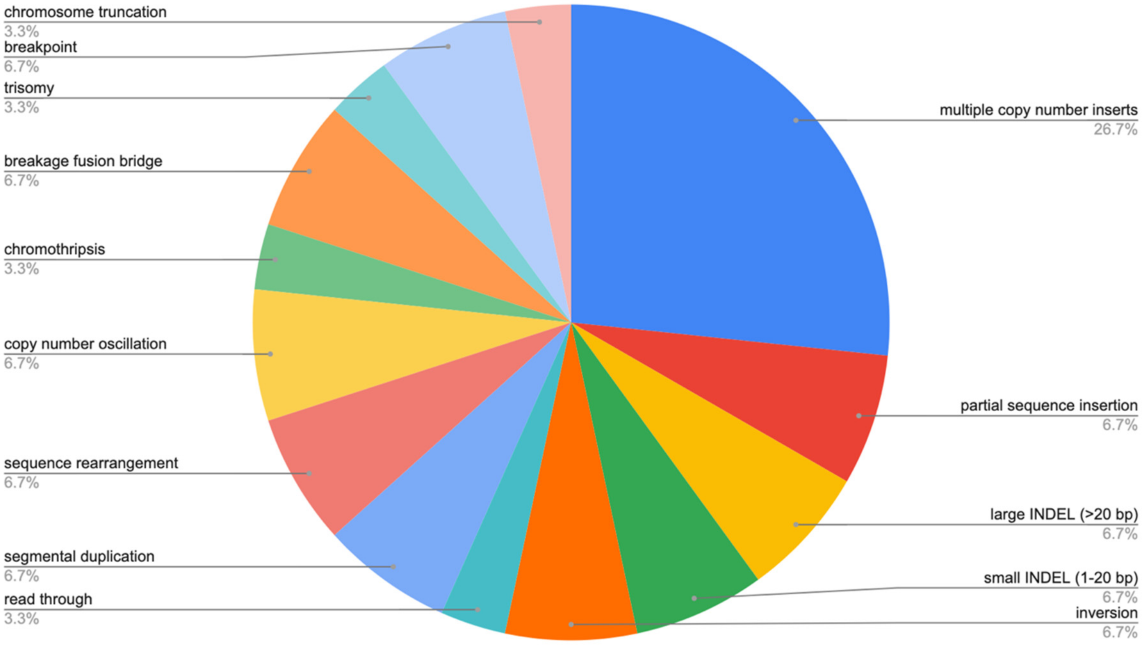

Figure S1. Genomic outcomes from Biolistic bombardment

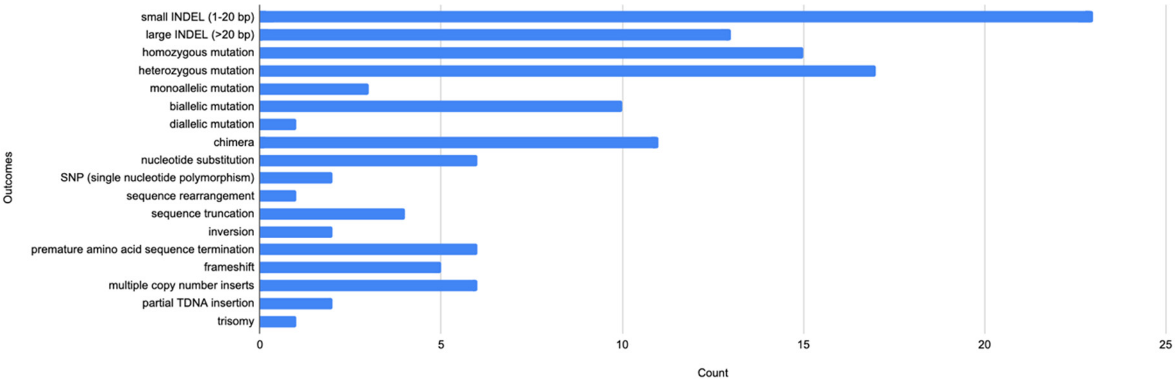

Figure S2. Genomic outcomes from CRISPR-Cas9 via *Agrobacterium*

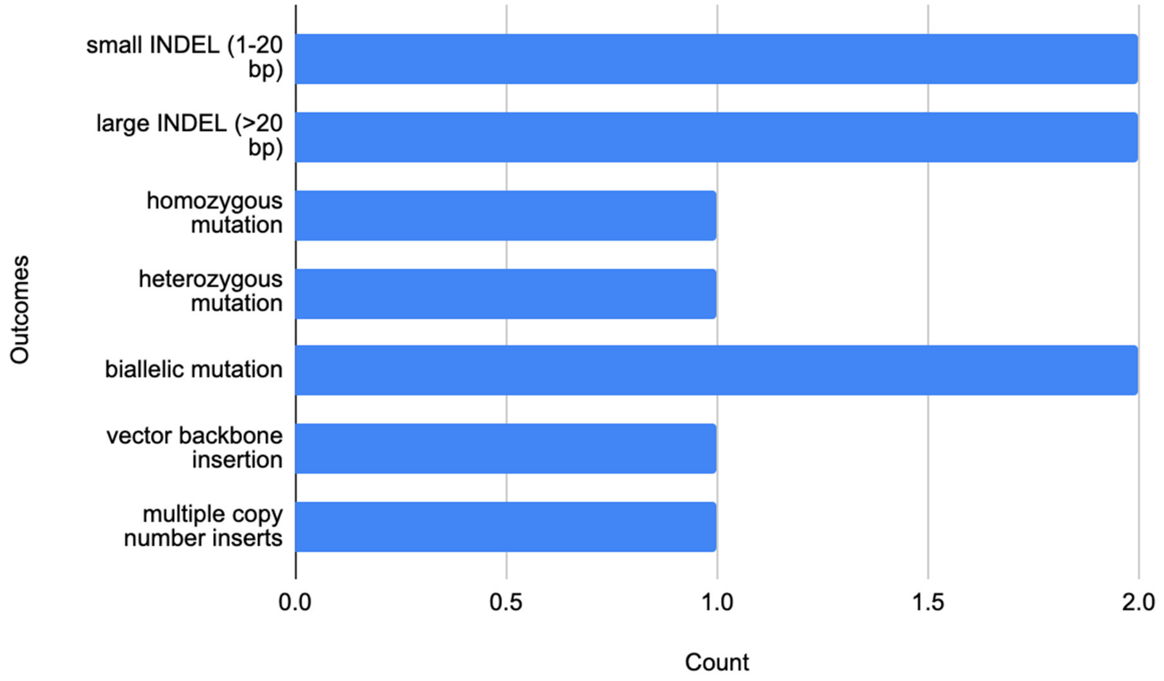

Figure S3. Genomic outcomes from CRISPR-Cas9 delivered via biolistic bombardment

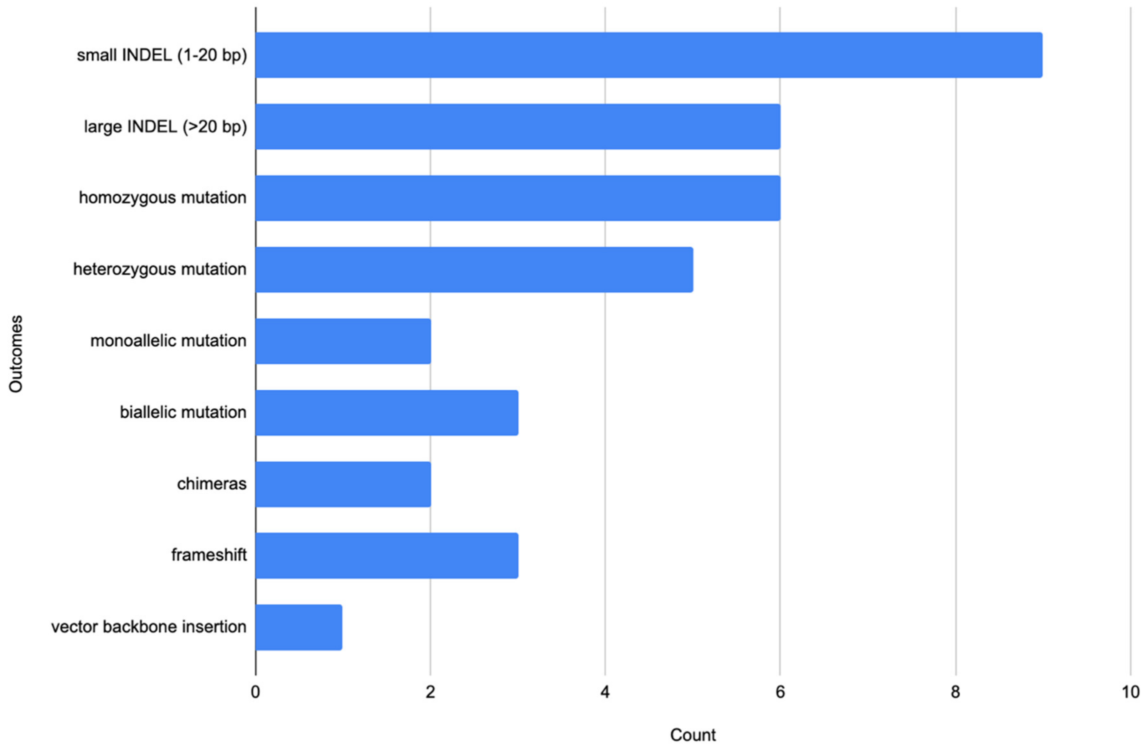

Figure S4. Genomic outcomes from CRISPR-Cas9 delivered as RNPs. The vector backbone insertion came from a paper that performed RNP/plasmid DNA co-delivery.
